# Prevalence of *Echinococcus* Species in Wild Foxes and Stray Dogs in Qinghai Province, China

**DOI:** 10.4269/ajtmh.21-0622

**Published:** 2021-11-15

**Authors:** Huixia Cai, Jing Zhang, Xuefei Zhang, Yayi Guan, Xiao Ma, Jianping Cao, Junying Ma, Na Liu, Hao Wu, Yufang Liu, Jia Liu, Wei Wang, Wen Lei, Kemei Shi, Qing Zhang, Xiongying Zhang, Peizhen Zhan, Yujuan Shen

**Affiliations:** ^1^ Department of Parasite Control, Qinghai Province Institute for Endemic Diseases Prevention and Control, Xining, China;; ^2^ National Institute of Parasitic Diseases, Chinese Center for Disease Control and Prevention (Chinese Center for Tropical Diseases Research), NHC Key Laboratory of Parasite and Vector Biology, WHO Collaborating Center for Tropical Diseases, National Center for International Research on Tropical Diseases, Shanghai, China;; ^3^ Department of Medical Record Information, Qinghai Provincial Traffic Hospital, Xining, China

## Abstract

Echinococcosis is a zoonotic parasitic disease that is highly endemic to the Qinghai province of China. Limited data are available on the prevalence of the causal pathogen, *Echinococcus* spp., in definitive hosts in this region. Thus, the aim of this study was to evaluate the prevalence of *Echinococcus* spp. in wild foxes and stray dogs in Qinghai province. Five hundred and twenty-eight feces from wild foxes and 277 from stray dogs were collected from 11 counties in the Golog, Yushu, and Haixi prefectures and screened for *Echinococcus* spp. using copro-DNA polymerase chain reaction (PCR). In total, 5.5% of wild foxes and 15.2% of stray dogs tested positive for *Echinococcus* spp. The prevalence rates of *Echinococcus* spp. in wild foxes in Golog, Yushu, and Haixi were 7.3%, 5.2%, and 1.9%, respectively. In stray dogs, these rates were 13.3%, 17.3%, and 0%, respectively. Sequencing analysis determined that *Echinococcus multilocularis* was the most prevalent species, occurring in 4.0% and 12.6% of wild foxes and stray dogs, respectively. *Echinococcus shiquicus* was observed in 1.5% of wild foxes and 0.7% of stray dogs. *Echinococcus granulosus* was observed only in wild dogs, with a prevalence rate of 1.8%. To our knowledge, this is the first report on the prevalence of *E. shiquicus* in dogs in Qinghai province. The current results improve our understanding of the transmission and dissemination of human echinococcosis and suggest that exposure to the eggs of *E. multilocularis* harbored by wild foxes and stray dogs may pose a great risk of alveolar echinococcosis to humans in Qinghai province.

## INTRODUCTION

Echinococcosis, caused by metacestodes of the genus *Echinococcus,* is a chronic zoonotic disease endemic around world.^1^ In the most recent taxonomic revision, nine species were recognized in the genus *Echinococcus*, including *Echinococcus granulosus* sensu stricto (Eg; genotypes G1–G3), *E. multilocularis* (Em), *E. oligarthra, E. vogeli, E. shiquicus* (Es), *E. equinus* (G4),* E. ortleppi* (G5), *E. canadensis* (G6–G10) (Ec), and *E. felidis*.^2^ Eg, Em, Ec, and Es have been isolated from animal hosts in China, whereas Eg, Em, and Ec have been detected in humans.^3^^,^^4^ Eg and Em cause cystic echinococcosis (CE) and alveolar echinococcosis (AE), respectively, in humans. Cystic echinococcosis and AE are significant threats to public health in the pastoral areas of western and northwestern China.^5^

Qinghai Province is one of the areas within China where echinococcosis (CE and AE) is highly endemic.^6^^,^^7^ The Golog Tibetan Autonomous Prefecture (Golog) and Yushu Tibetan Autonomous Prefecture (Yushu) of the southern plateau region in Qinghai are the most severely affected, with echinococcosis prevalence ranging from 0.2% to 12.38% for 12 Tibetan counties across these prefecture in 2012.^8^ Further, the Golog had the highest average prevalence of human echinococcosis (5.2%),^9^ whereas in Yushu it was 4.54%.^10^ The prevalence ranges for counties within these two Tibetan autonomous prefectures were 0.2–8.2% (AE) and 2.62–6.11%(CE), respectively.^11^^–^^16^ However, the Haixi Menggu and Tibetan Autonomous Prefecture (Haixi), neighbors of Yushu and Golog, exhibited relatively low echinococcosis prevalence, and only CE has been reported there.^17^

Echinococcosis is transmitted via carnivore definitive and herbivore/omnivore intermediate hosts through predator–prey interactions, which results in different life cycles types, such as domestic, wildlife, or hybrid. Eg is predominately transmitted among dogs and livestock, whereas the transmission cycles of Em and Es involve dogs and wild foxes as the primary definitive hosts, and a variety of small mammals as intermediate hosts. Humans are typically infected with *Echinococcus* spp. through direct contact with feces from infected animals, or via the consumption of contaminated food and water. In Tibetan areas, domestic and stray dogs are the biggest contributors to CE and AE prevalence, whereas wild foxes are the primary source of AE.^18^^,^^19^ In recent years, there have been more investigations on domestic dogs,^9^^,^^10^^,^^14^^,^^20^ and limited research on nondomesticated carnivores. Studying the latter group is critical for understanding the risk posed to humans.

In the present study, we investigated the prevalence of *Echinococcus* spp. in wild foxes and stray dogs in Golog, Yushu, and Haixi prefectures of Qinghai province using copro-DNA polymerase chain reaction (PCR) analysis. The current findings will contribute to the development of prevention and control strategies for echinococcosis in these areas.

## MATERIALS AND METHODS

### Study regions.

**Figure 1.**
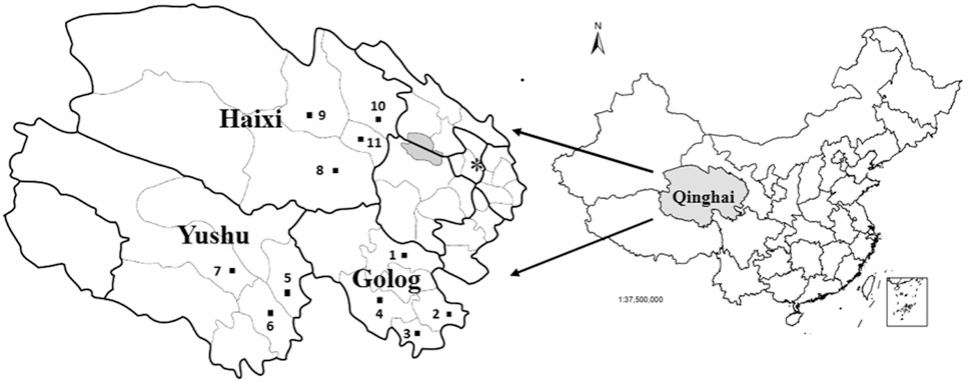
Study areas in Qinghai province, China. *Xining, provincial capital; sites 1–11, samples collected from 11 counties across three prefectures.

Study regions were selected based on well-documented high prevalence and geography. The regions evaluated in this study are shown in Figure 1.

Golog (longitude: 97°54′–120°50′; latitude: 32°31′–35°40′) is a pastureland area in the Qinghai-Tibet Plateau, with a mean altitude of 4,200 m above sea level, and an annual mean temperature of 1.1°C. There are a large number of canids, including domestic and stray dogs, foxes, and wolves, as well as > 250,000 livestock. Fecal samples were collected from four counties (Maqin, Dari, Jiuzhi, and Banma) in 2015.

Yushu (longitude: 89°27′–97°39′; latitude: 31°45′–36°10′) is a pastureland area located in the Qinghai-Tibet Plateau, adjacent to Haixi and Golog. Similar to Golog, Yushu has a mean altitude of 4,200 m above sea level, with an annual mean temperature of 3.3°C. Large numbers of livestock and wild canids inhabit this area. Fecal samples were collected from three counties (Yushu, Chenduo, and Zhiduo) in 2014.

Haixi is located in the west of Qinghai province and north of the Qinghai-Tibet Plateau (longitude: 90°06′–99°42′; latitude: 35°01′–39°20′), at the southern border of Golog and Yushu. It comprises part of the Qaidam Basin, most of the Gobi Desert, marshes, and saltwater lakes, with a terrain typical of the highest plateau inland basins. The mean altitude of the basins is 3,000 m above sea level, and the annual mean temperature is 5.2°C, with a continental arid climate. The economy of Haixi drives by industries, with a minor contribution from agriculture and animal husbandry. Fecal samples were collected from four counties (Delinha, Wulan, Dulan, and Tianjun) in 2015.

### Specimen collection.

In the 11 investigated counties, we determined a collection area at every 2 km or more along the road in each county, depending on the surrounding environment, the distribution of rat holes, the haunt of the foxes and stray dogs, and so on. At least a dozen such collection areas were designated in each county. Wild fox feces were identified by shape, color, moisture, and smell, and were collected from rodent holes/mounds, soddy walls, and ditches. Stray dog feces were collected from inhabited areas of the Golog and Yushu prefectures, but not from Haixi, where stray dogs are extremely rare. To minimize the repeated sampling of feces from the same animal, each samples was collected at a minimal distance of 200–300 m from the previous. Each sample was individually collected in a 50-mL centrifuge tube and stored at −80°C for at least 1 week to inactivate the eggs of *Echinococcus* spp.^21^

### DNA extraction and host species identification.

Total genomic DNA was extracted from fecal samples (200–300 mg) using the QIAamp DNA Stool Mini Kit (Qiagen, Hilden, Germany) according to the manufacturer’s instructions. The inhibitEX tablets and Buffer ASL from the Kit efficiently adsorbed substances in the fecal samples that may degrade DNA from the fecal samples. The extracted genomic DNA samples were stored at −20°C. To identify the origin species in the fecal samples, the primer pair H15149L/L14724 was used to amplify cytochrome b (729 bp) mitochondrial DNA of the family Canidae via copro-DNA PCR, as previously described.^22^ All DNA samples were amplified at least three times. All PCR products were sequenced by Shanghai Biotechnology Co. (Shanghai, China) and compared against sequences in the NCBI database. Only DNA samples extracted from foxes and dogs were used for *Echinococcus* spp. identification.

### Parasite identification.

Em and Es were identified using nested-PCR for the mitochondrial cytochrome c oxidase subunit I (*coxI*) gene,^23^ and Eg was identified using species-specific primers for the mitochondrial NADH dehydrogenase subunit I (*nadI*) by PCR, as previously described.^24^^–^^27^ Polymerase chain reaction protocols are described in Table 1. For all PCRs, distilled water was used as a negative control, and DNA from the adult worms of Em, Es, and Eg was used as positive controls. Each DNA sample was amplified at least three times. PCR products were visualized using 1.5% agarose gel electrophoresis with ethidium bromide staining. Positive PCR products were sequenced by Shanghai Biotechnology Co. Each sequence was compared against sequences in the NCBI database to determine the species of *Echinococcus* spp.

**Table 1:** Summary of PCR protocols for species identification

Target	Gene	Step	Primers	Product (bp)	Method
Taeniidae^25^	*CoxI* (external)	First	FP:5’TTGAATTTGCCACGTTTGAATGC-3’ RP:5’GAACCTAACGACATAACATAATGA-3’	874	Nested-PCR
*Echinococcus multilocularis* ^26^	*CoxI* (internal)	Second	FP:5’GTCATATTTGTTTAAGTATAAGTGG-3’ RP:5’CACTCTTATTTACACTAGAATTAAG-3’	243	
*Echinococcus shiquicus* ^27^	*CoxI* (internal)	Second	FP:5’GTTGGTTACGTTACCGGTT-3’ RP:5’-TCTTATTAACATTTGAATTCAAC-3’	420	
*Echinococcus granulosus* ^24^	*NadI*	First	FP:5’GGTTTTATCGGTATGTTGGTGTTAGTG-3’ RP:5’CATTTCTTGAAGTTAACAGCATCACG-3’	219	Ordinary PCR

PCR = polymerase chain reaction.

### Statistical analysis.

Data were analyzed using SPSS software (SPSS Inc., Chicago, IL) and mapped using ArcGIS 10.1 (ESRI, RedLands). Differences in the presence of *Echinococcus* spp. were evaluated with the χ^2^ test. *P* < 0.05 was considered to indicate statistically significance. The Wilson score method was used to calculate the 95% CI for each group of proportions.

## RESULTS

### Prevalence of *Echinococcus* spp. in wild foxes and stray dogs.

**Figure 2.**
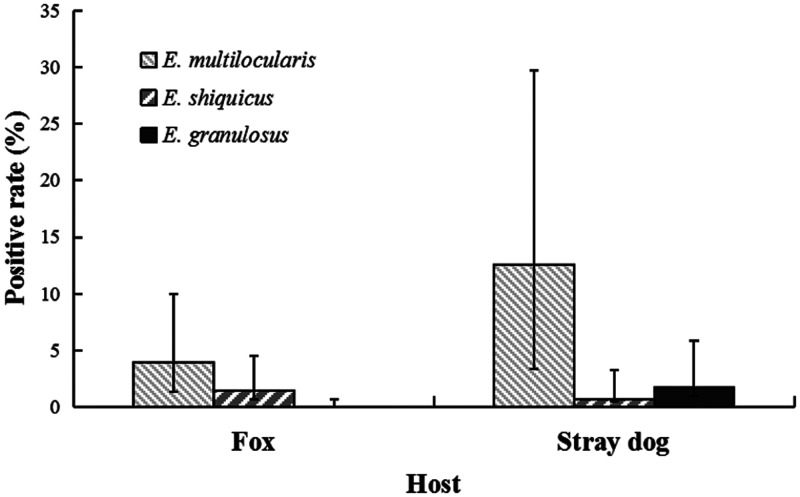
Prevalence of *Echinococcus* species in fecal samples from wild foxes and stray dogs.

Of the 805 collected fecal samples, 528 were from wild foxes, and 277 were from stray dogs. Twenty-nine samples from wild foxes were positive for *Echinococcus* spp. (5.5%, 95% CI: 3.9–7.8). Among these, 21 contained Em (4.0%, 95% CI: 2.6–6.0), and eight contained Es (1.5%, 95% CI: 0.8–3.0). Forty-two stray dogs were positive for *Echinococcus* spp., with a prevalence of 15.2% (95% CI: 11.4‒19.9, 42/277). Of these, 35 contained Em (12.6%, 95% CI: 9.2–17.1), two contained Es (0.7%, 95% CI: 0.2–2.6), and five contained Eg (1.8%, 95% CI: 0.8–4.1). All Eg-positive samples were identified as of the G1 genotype, based on sequencing of the *nadI* gene. The general prevalence rates of *Echinococcus* spp. (χ^2^ = 21.13, *P* < 0.001) and the prevalence of specific *Echinococcus* species (χ^2^ = 31.89, *P* < 0.001) differed significantly between the two hosts (Figure 2). No animals were infected by more than one *Echinococcus* species.

### Prevalence of *Echinococcus* spp. by geographic region.

In Golog, 246 wild fox fecal samples and 150 stray dog fecal samples were collected. In Yushu, 174 wild fox fecal samples and 127 stray dog fecal samples were collected. About 108 wild fox fecal samples were collected from Haixi. The prevalence rate of *Echinococcus* spp. in wild foxes was 7.3% in Golog, 5.2% in Yushu, and 1.9% in Haixi, differing significantly among the three prefectures (χ^2^ = 11.49, *P* < 0.05). The prevalence rates of *Echinococcus* spp. among stray dogs were 13.3%and 17.3% in Golog and Yushu, respectively, with no significant differences between the prefectures (χ^2^ = 5.43, *P* > 0.05). Significant differences in the prevalence of *Echinococcus* spp. were observed between wild foxes and stray dogs in Yushu (χ^2^ = 20.91, *P* < 0.001) and in Golog (χ^2^ = 7.81, *P* < 0.05).

In Golog, 16 (6.5%) wild fox fecal samples were positive for Em and two (0.8%) for Es. Of the nine fecal samples from wild foxes in Yushu, which were positive for *Echinococcus* spp., five (2.9%) contained Em, and four (2.3%) contained Es. In Haixi, two *Echinococcus* spp.-positive wild fox fecal samples (1.9%) contained Es, while Em was not detected. None of the fecal samples from wild foxes collected across the three prefectures tested positive for Eg. The prevalence rate of Es among wild fox fecal samples was not significantly different across the three prefectures (χ^2^ = 1.61, *P* > 0.05). Em occurrence rates among wild fox fecal samples were significantly different between Golog and Haixi (χ^2^ = 7.36, *P* < 0.01). In addition, Em was identified in 9.3% (14/150) and 16.5% (21/127) of fecal samples from stray dogs in Golog and Yushu, respectively, and these rates were not significantly different (χ^2^ = 3.23, *P* > 0.05). Furthermore, four stray dog fecal samples from Golog (4/150, 2.7%) and one from Yushu (1/127, 0.8%) were positive for Eg (not significantly different: χ^2^ = 1.37, *P* > 0.05). Es was also detected in stray dog fecal samples from Golog (2/150, 1.3%) (Table 2).

**Table 2:** Prevalence of *Echinococcus* species in wild foxes and stray dogs in Qinghai province

Host	Location	Number of samples	Number of positive samples (%/95% CI)			Total number of positive samples
			*Echinococcus multilocularis*	*Echinococcus shiquicus*	*Echinococcus granulosus*	(%/95% CI)
Fox	Golog	246	16 (6.5/4.0–10.3)	2 (0.8/0.2–2.9)	0 (0/0.0–1.5)	18 (7.3/4.7–11.3)
	Haixi	108	0 (0/0.0–3.4)	2 (1.9/0.5–6.5)	0 (0/0.0–3.4)	2 (1.9/0.5–6.5)
	Yushu	174	5 (2.9/1.2–6.6)	4 (2.3/0.9–5.8)	0 (0/0.0–2.16)	9 (5.2/2.7–9.5)
Stray dog	Golog	150	14 (9.3/5.6–15.1)	2 (1.3/0.4–4.7)	4 (2.7/1.0–6.7)	20 (13.3/8.8–19.7)
	Yushu	127	21 (16.5/11.1–24.0)	0 (0/0.0–2.9)	1 (0.8/0.1–4.3)	22 (17.3/11.7–24.8)

## DISCUSSION

The prevention and control of echinococcosis in Qinghai province is a long-term and arduous task. Echinococcosis was prevalent in 39 out of 43 counties in Qinghai Province, covering a total area of 650,000 km^2^ with AE and CE coexisting in 14 counties. The most severely affected area is the southern part of Qinghai province, where the population density is 1.8 person/km^2^. These area located in high-altitude pastures, where local residents engage in semi-nomadic livestock production activities, thus being more closer to domestic and wild canids and therefore are at a greater risk of contracting echinococcosis.

Dogs were identified as a major source of human echinococcosis infection in a previous study.^28^ Twenty years ago, the prevalence rate of *Echinococcus* spp. among domestic dogs in Qinghai province was 40%, with rates up to 70% in Golog and Yushu.^29^ In 2006–2015, the National Echinococcosis Control Project adopted a program for deworming domestic dogs (using praziquantel) as a substantial control measure, that was incrementally carry out from three to 39 counties in Qinghai province. In 2012, the prevalence of *Echinococcus* infection among domestic dogs was 13.02%.^8^ Thus, the prevalence decreased significantly from 40% to 13.02%, owing to the above-described deworming strategy. In 2010, the implementation of the National Echinococcosis Control Project has gradually expanded from a single health department to 14 departments including agriculture, forestry and grassland, water resources and public security, and so on.^30^ Since then, endemic counties across Qinghai have started to implement measures such as domestic dog registration and management, partial-stray dog aperiodic reception or disposal, livestock immunization and slaughter management, grassland management, rodent control, and so on. In fact, 10 years ago, stray dogs and unleashed domestic dogs were ubiquitous in all counties of Qinghai Province. Since 2010, the number of stray dogs has been significantly reduced. This was also reflected by our study, where the number of fecal samples collected from stray dogs was less than that from the foxes. It is worth noting that the wild foxes and stray dogs could still be observed in and around human settlements during our survey in 2014–2015, although wild foxes is common far from villages and the some stray dog were also adopted or disposed irregularly. Their feces, which contain eggs, can therefore contaminate surroundings, posing a significant risk of infection to local residents, herders, or travelers.

Control programs have certainly had an impact on the prevalence of *Echinococcus* in stray dogs and wild foxes. Compared with data of stray dogs in the Qinghai southern plateau from studies conducted during the 1995–2010 period,^31^ the prevalence rates of Em and Eg among stray dogs in Golog and Yushu determined in the current study were lower. Furthermore, the prevalence rates of Em in wild foxes were lower compared with data of wild foxes in Qinghai southern plateau. There may be various reasons for this difference, including deworming measure, the prohibition of unleashed domestic dogs, as well as the implementation of grassland rodent and partial-stray dog aperiodic reception or disposal measures. Our study found that the prevalence of *Echinococcus* in stray dogs was higher than wild foxes, which was in agreement with a report from 2018.^32^ Further, the prevalence of *Echinococcus* in stray dogs was similar to that in domestic dogs in Qinghai Province (13.02%). These data indicate that stray dogs are the major source of infection in the wildlife transmission cycle, followed by wild foxes. The level of *Echinococcus* infection in stray dogs is similar to that in domestic dogs, suggesting that both represent important targets for echinococcosis control in the future. In the current study, the prevalence of Eg in stray dogs was significantly lower than Em. The Eg infection prevalence among stray dogs in Golog and Yushu was lower than that of previously reports for the Qinghai southern plateau, possibly because of the recent enforcement of livestock slaughter regulations. This further suggests that stray dogs might feed on predatory wild rodents rather than on the abandoned organs of slaughtered cattle or sheep from human settlements. Thus we presume that compared with wild foxes, stray dogs may pose a greater risk to humans with regard to AE and should thus be the focus of future transmission prevention and control within wildlife cycles. Meanwhile, we should not neglect the role of wild foxes in Em transmission. In Haixi, animal husbandry is a relatively small contributor to the economy, and fewer stray dogs are present. Previous studies identified Haixi as a region of low CE prevalence, with a complete absence of AE.^17^ Thus, no fecal samples were collected from stray dogs in this area, which may be one reason for the low prevalence of echinococcosis. Further, we did not observe Em in wild foxes from Haixi.

Several Es-positive samples from wild foxes were identified in all three regions. It is worth noting that Es was also detected in two stray dogs from Dari county in Golog, consistent with a previous study conducted in Sichuan.^33^ Interestingly, this pre-dates the detection of Em in plateau pika (*Ochotona curzoniae*) in Golog.^11^^,^^12^^,^^34^ To our knowledge, the current work represents the first report of Es in dogs from Qinghai province. Es is generally considered nonpathogenic to humans, and the epidemiological significance of dogs as definitive hosts remains to be determined.^26^ Eg was not observed in wild fox fecal samples, which is consistent with a study conducted in Shiqu county, Sichuan province.^23^ In Australia, Eg has been reported to infect red foxes.^35^ However, foxes are not considered definitive hosts of Eg in China.^5^ In fact, the infection rates of parasites based on fecal samples are influenced by various factors, including the sampling locus, sample status, and detection methods. Nonetheless, positivity rates can still reflect the prevalence and distribution characteristics of parasitic diseases.

We reviewed literature to understand the prevalence of *Echinococcus* spp. worldwide and have summarized the findings in Table 3. The overall prevalence of *Echinococcus* spp. in definitive hosts varies globally. In China, the prevalence of *Echinococcus* spp. in domestic dogs has received considerable attention, but there have only been a few recent studies on *Echinococcus* spp. infection in wild foxes and stray dogs, which is of equal importance for assessing the risk of transmission to humans. Furthermore, previous studies largely focused on comparisons between detection methods, or are limited by small sample sizes for species identification.^23^^–^^25^^,^^33^ Therefore, systematic epidemiological studies for the prevalence of *Echinococcus* spp. in wild canines are of great importance. In the current study, the prevalence of *Echinococcus* species in wild foxes and stray dogs was determined, providing an overview of prevalence rates in wild canines across various endemic regions throughout Qinghai province. Consequently, our results provide valuable insight into *Echinococcus* infection in wild foxes and stray dogs in highly endemic area of China as well as a basis for risk assessment and the development of control and prevention methods.

**Table 3:** Worldwide prevalence of *Echinococcus* spp. in foxes and dogs over the last 3 years

Country	Location	Host	Positive no./sample no. (%)	*Echinococcus* species (no. positive samples)
China	Ningxia^36^	Domestic dog	250/750 (33.3)	Em (106); Eg (124); mixed Em and Eg (20)
	Qinghai*	Wild fox	29/528 (5.5)	Em (21); Es (8)
		Stray dog	42/277 (15.2)	Em (35); Es (2); Eg (5)
	Qinghai^20^	Domestic dog	36/144 (25.0)	Eg (35); mixed Em and Eg (1)
	Qinghai^32^	Wild fox	6/161 (3.7)	–
		Stray dog	8/61 (13.1)	–
	Sichuan^37^	Domestic dog	11/120 (9.2)	Em
	Tibet^38^	Domestic dog	552/7,564 (7.3)	–
	Xinjiang^39^	Domestic dog	74/2,219 (3.3)	–
Bhutan	Tsirang/Gelephu^40^	Stray dog	10/138 (7.3)	Eg
Canada	Ontario^41^	Wild fox	9/44 (20.5)	Em
France	Corsica^42^	Domestic dog	3/259 (1.2)	Ec
India	Maharashtra^43^	Stray/domestic dog	19/289 (6.6)	–
Iran	Kerman^44^	Stray dog	34/307 (11.1)	Eg (21); others (13)
Kenya	Turkana/Isiolo/Meru/ Narok^45^	Domestic dog	71/1,621 (4.4)	Eg (43); Ec (15); Eo (4); mixed Eg and Ec (7); mixed Eg and Eo (1); mixed Eg, Ec and Eo (1)
Poland	Podkarpackie^46^	Domestic dog	4/268 (1.5)	Em
		Wild fox	53/110 (48.2)	Em
Serbia	Vojvodina^47^	Wild fox	29/223 (13.0)	Em
Sudan	Khartoum^48^	Stray dog	40/84 (47.6)	Ec (39); Eg (1)
Uzbekistan	Samarkand^49^	Wild fox	0/5 (0)	–
		Domestic dog	24/1,749 (1.4)	Eg
		Stray dog	4/6 (66.7)	Eg

Em = *Echinococcus multiloculosus*; Ec = *Echinococcu canadensis*; Eg = *Echinococcu granulosus*; Es = *Echinococcu shiquicus*; Eo = *Echinococcus ortleppi*.

* This study. – indicates no *Echinococcus* species test.

## CONCLUSION

Our results demonstrate that Qinghai province has a high prevalence of *Echinococcus* spp. (specifically, Em, Es, and Eg) in wild foxes and stray dogs. These findings are crucial for the control and prevention of echinococcosis in the region. Further molecular characterization is essential to better understand the distribution and diversity of *Echinococcus* spp.
